# Reduction in Pathogen Populations at Grapevine Wound Sites is Associated with the Mechanism Underlying the Biological Control of Crown Gall by *Rhizobium vitis* Strain ARK-1

**DOI:** 10.1264/jsme2.ME14059

**Published:** 2014-07-31

**Authors:** Akira Kawaguchi

**Affiliations:** 1 Research Institute for Agriculture, Okayama Prefectural Technology Center for Agriculture, Forestry and Fisheries, 1174–1 Koudaoki, Akaiwa City, Okayama 709–0801, Japan

**Keywords:** *Rhizobium vitis*, grapevine crown gall, biological control, pathogen populations, antibiosis assay

## Abstract

A nonpathogenic strain of *Rhizobium* (=*Agrobacterium*) *vitis*, ARK-1, limited the development of grapevine crown gall. A co-inoculation with ARK-1 and the tumorigenic strain VAT07-1 at a 1:1 cell ratio resulted in a higher population of ARK-1 than VAT07-1 in shoots without tumors, but a significantly lower population of ARK-1 than VAT07-1 in grapevine shoots with tumors. ARK-1 began to significantly suppress the VAT07-1 population 2 d after the inoculation. This result indicated that ARK-1 reduced the pathogen population at the wound site through biological control. Although ARK-1 produced a zone of inhibition against other tumorigenic *Rhizobium* spp. in *in vitro* assays, antibiosis depended on the culture medium. ARK-1 did not inhibit the growth of tumorigenic *R. radiobacter* strain AtC1 in the antibiosis assay, but suppressed the AtC1-induced formation of tumors on grapevine shoots, suggesting that antibiosis by ARK-1 may not be the main mechanism responsible for biological control.

Grapevine (*Vitis vinifera* L.) crown gall, which is mainly caused by *Rhizobium vitis* (Ti) (40) [=*Agrobacterium vitis* (Ti), *A. tumefaciens* biovar 3, where “Ti” means “tumor-inducing” or “tumorigenic”], is one of the most important diseases of grapevines worldwide (7, 8, 36). The pathogenicity genes are mostly located on large tumor-inducing plasmids (pTi). During infection, part of the plasmid (T-DNA) is transferred to the host plant and inserted into its nuclear DNA (11). The main symptom of crown gall is the fleshly galls that are produced in response to infection (4). These galls are mostly found on the lower trunk of grapevines near the soil surface (4). Crown gall represents a serious economic problem in viticultural regions of northern Japan, in which *Vitis vinifera* and interspecific hybrids are grown and climatic conditions favor freeze injury. The incidence of crown gall was previously reported to be high at the basal ends and belowground disbudded nodes of some cultivars and rootstocks in nurseries in Japan. Shoot growth by galled vines is frequently inferior, and portions of the vine above these galls can die (4). Thus, grapevine crown gall causes substantial economic losses, and there is currently no effective strategy for managing this disease.

The nonpathogenic *R. rhizogenes* strain K84 has successfully been used to control crown gall in many plant species (14, 28, 30, 33). An agrocin produced by K84 (agrocin 84) is thought to be the primary factor responsible for this control (28). However, K84 does not control grapevine crown gall caused by *R. vitis* (Ti), which is insensitive to agrocin 84 (3, 5, 18, 19, 21). Recombinant DNA techniques have been used to construct the new biological control strain, K1026, which is identical to K84 except for a 5.9-kb deletion that overlaps the transfer (Tra) region of pAgK84 (16). K1026 is unable to transfer its mutant agrocin 84 plasmid, designated pAgK1026, to other agrobacteria, but remains inhibitory to strains that are sensitive to agrocin 84 (16, 33, 34).

Several laboratories have attempted to identify other biological measures to control grapevine crown gall (10, 13, 38, 39). Staphorst *et al.* (38) evaluated nonpathogenic *R. vitis* strain F2/5, which inhibited the growth of most tumor-inducing strains of *R. vitis in vitro* and greatly inhibited crown gall formation on grapevines in greenhouse shoot-wounding experiments. Burr and Reid (6) reported that F2/5 produced agrocin, which inhibits most *R. vitis* (Ti) strains *in vitro*, and effectively inhibited tumor formation at wound sites on grapevine shoots artificially inoculated with one of several *R. vitis* (Ti) strains. However, F2/5 did not inhibit tumor formation caused by other strains of *R. vitis* (Ti) (8), and F2/5 caused necrosis on grapevine shoot explants (15). Wang *et al.* (39) reported that the antibacterial compound Ar26 produced by nonpathogenic *R. vitis* strain E26 inhibited the growth of *R. vitis* (Ti) strain MI3-2 and *R. radiobacter* (Ti) strain CY4 on culture plates. Chen *et al.* (10) also demonstrated that *Rahnella aquatilis* strain HX2 inhibited the development of crown galls on grapevines. Although these antagonistic strains have been described in the literature, they have not yet been implemented.

We previously reported that the nonpathogenic *R. vitis* strain, VAR03-1, which was isolated from a grapevine nursery stock in Japan, inhibited tumor formation caused by several *R. radiobacter* (Ti), *R. rhizogenes* (Ti), and *R. vitis* (Ti) strains isolated from different plants in Japan on grapevines, roses, tomatoes, sunflowers, and apples (18, 19, 21, 22, 23, 25). Repetitive sequence-based polymerase chain reaction (rep-PCR) DNA fingerprint analysis and an analysis of the sequences of three housekeeping genes revealed that VAR03-1 differed genetically from the tumorigenic and non-antagonistic *R. vitis* strains (21, 26). Moreover, nonpathogenic *R. vitis* strain ARK-1, which was found to be more effective than VAR03-1 at inhibiting tumor formation on grapevines, was identified as a new antagonistic strain (24). ARK-1 is endophytic in grapevines, and treatments with ARK-1 controlled grapevine crown gall better than VAR03-1 in field trials (27). We have been developing a new bactericide of ARK-1 to control grapevine crown gall together with a private enterprise. ARK-1 did not produce a zone of inhibition around the *R. vitis* (Ti) strain on yeast-mannitol agar (YMA) medium, and did not reduce the incidence of tumors in grapevine shoots when it was killed by autoclaving or when the culture filtrate alone was used, indicating the absence of some bacteriocins or antibacterial materials in the dead-cell suspension and culture filtrate of ARK-1 (24). These findings indicated that ARK-1 inhibited grapevine crown gall in plants by a different mechanism from that of VAR03-1 (24).

Thus, we hypothesized that ARK-1 could not inhibit the growth of tumorigenic strains on culture plates, but could on grapevines. Moreover, we attempted to confirm whether ARK-1 did not produce bacteriocins or antibacterial materials on any kinds of culture plates. We herein demonstrated that a reduction in the pathogen population at the grapevine wound site by ARK-1 was responsible for the biological control achieved by this strain, and also that antibiotic activity was not the main control mechanism. In the present study, we followed the nomenclature for *Rhizobium* species adopted by Young *et al.* (40) to avoid confusion, although other valid naming systems have been proposed (1, 9, 29, 31, 32, 37, 41).

## Materials and Methods

### Development of antibiotic resistant *R. vitis* strains

The antibiotic-resistant mutants of two strains, ARK-1sc and VAT07-1n, were used in a survival assay to differentiate the inoculated biological control agents from indigenous agrobacteria. Potato sucrose agar (PSA: 300 g potato, 0.5 g Ca[NO_3_]_2_·4H_2_O, 2 g Na_2_HPO_4_·12H_2_O, 5 g peptone, 20 g sucrose, 15 g agar, 1 L distilled water, pH 6.8–7.0) medium was used to grow the bacteria in this study. ARK-1sc was a streptomycin (St)-copper sulfate (CuSO_4_)-resistant mutant (St-CuSO_4_-mutant) obtained by growing strain ARK-1 on St-CuSO_4_-PSA medium, which is PSA amended with 500 ppm St and 250 ppm CuSO_4_ (24, 27). VAT07-1n was a nalidixic acid (Nal)-resistant mutant (Nal-mutant) obtained by growing strain VAT07-1 on Nal-PSA medium, which is PSA amended with 50 ppm Nal. VAT07-1 was grown on Nal-PSA by selecting the colonies that had wild-type growth rates on PSA medium with no antibiosis. This method was repeated twice to confirm the stability of Nal resistance. The cell suspensions of ARK-1sc and VAT07-1n were prepared from 48-h-old cultures on PSA slants and adjusted to OD_600_ = 0.1 (corresponding to approximately 10^8^ cells mL^−1^) and mixed at cell ratios of 1:1.

### Survival of ARK-1sc and VAT07-1n on grapevine seedlings

Grapevine seedlings (*V. vinifera* cv. ‘Neo Muscat’) were grown from seeds. One-year-old grapevine shoots were inoculated using previously established methods (18, 19). An outline of this experiment is shown in Fig. S1. A 5-μL drop of a cell suspension (ARK-1sc, VAT07-1n, or mixed) was dropped onto a needle-prick wound on the grapevine shoot. Each grapevine seedling (*n* = 30, one plant per pot) was inoculated once with ARK-1sc or VAT07-1n in each treatment, and each grapevine seedling (*n* = 60, one plant per pot) was inoculated once with the mixed cell suspension of ARK-1sc and VAT07-1n in each treatment. Each grapevine seedling represented one biological replicate. The seedlings were grown in a greenhouse at 20 to 35°C for 3 months with natural sunlight. Tumors developed in 30 plants inoculated with VAT07-1n and in 10 plants co-inoculated with the mixed cell suspension of ARK-1sc and VAT07-1n, whereas no tumors developed in 30 plants inoculated with ARK-1sc pr in 50 plants co-inoculated with the mixed cell suspension. To determine the populations of each strain, 10 shoots were randomly sampled at the inoculation wound site (0.1 g fresh weight per plant, 1 sample per plant) 3 months after the inoculation from 30 plants inoculated with ARK-1sc and 60 plants co-inoculated with ARK-1sc and VAT07-1n. One tumor per plant (0.5 to 1.0 g fresh weight per plant) was randomly collected from 30 plants inoculated with VAT07-1n and from 60 plants co-inoculated with ARK-1sc and VAT07-1n. Each tumor was scrubbed by hand, rinsed under tap water for 10 s, and then blotted dry with paper towels. The surface of the tumor was washed with sterile distilled water, and then crushed in 1 mL of sterile distilled water with an autoclaved mortar and pestle. Ten-fold serial dilutions (100 μL) of the samples were then plated on St-CuSO_4_-PSA and Nal-PSA, and the plates were incubated at 25°C for 5 d. Colony growth was then observed on five plates for each dilution, and the numbers of colony-forming units (CFU) of strains ARK-1sc and VAT07-1n were counted on each medium. The bacterial populations in the wounded shoots and tumors (CFU g^−1^ of the grapevine shoot) were log_10_-transformed before statistical analysis. This assay was performed twice.

### Population dynamics of coexistence of ARK-1sc and VAT07-1n on grapevine seedlings

Grapevine seedlings (2 years old, ‘Neo Muscat’) were inoculated with ARK-1sc and VAT07-1n at a cell ratio of 1:1 as described above. An outline of this experiment is shown in Fig. S2. Each grapevine seedling (*n* = 65, one plant per pot) was inoculated once with the mixed cell suspension of ARK-1sc and VAT07-1n. Each grapevine seedling represented one replication. The seedlings were grown in a greenhouse at 20 to 35°C with natural sunlight. To determine the population dynamics of each strain, shoot samples including the wound site (0.2 g fresh weight per plant, 1 sample per plant) were collected from 5 plants (*i.e.*, *n* = 5) at 1, 2, 5, 9, 11, 14, 18, 23, 30, 37, 46, 63, and 88 d after the inoculation (assessed on 13 dates). After washing and crushing of the samples as described above, ten-fold serial dilutions (100 μL) were plated on St-CuSO_4_-PSA and Nal-PSA media. The colonies were incubated and counted as described above. This assay was performed twice.

### Dependence of tumor formation on the bacterial titer

The modeling experiments were performed to demonstrate the dependence of the incidence of tumors on variations in the titers of the pathogenic strain between 10^7^ and 10^6^ cells mL^−1^, and also to determine whether these differences significantly affected the rate of tumor induction. Concentrations of VAT07-1n were adjusted to 10^8^, 10^7^, 10^6^, and 10^5^ cells mL^−1^ based on OD_600_ values. Grapevine seedlings (2 years old, ‘Neo Muscat’) were inoculated with various concentrations of VAT07-1n as described above. Six pots of grapevine seedlings (one plant per pot) each received 10 inoculations (*i.e.*, a total of 60 inoculations). The seedlings were grown in a greenhouse at 20 to 35°C for 6 months with natural sunlight. The formation of tumors on the roots and stems of grapevines was assessed six months after the inoculation. This experiment was performed three times.

### *In vitro* antibiosis assay

The antibiosis assay was based on a previously described method (19, 20, 21, 25). A sterile paper disk (8 mm in diameter) with 50-μL of the ARK-1 cell suspension as the test strain (approximately 10^8^ cells mL^−1^) was placed on culture plates containing PSA medium; King’s B medium (10 g peptone, 1.5 g anhydrous K_2_HPO_4_, 15 g glycerol, 1.5 g MgSO_4_, 15 g agar, and 1 L distilled water, pH 7.0); YMA medium (0.4 g yeast extract, 10 g mannitol, 0.1 g NaCl, 0.2 g MgSO_4_, 0.5 g K_2_HPO_4_, 15 g agar, and 1 L distilled water, pH 7.0); nutrient agar (NA) medium (5 g peptone, 3 g yeast extract, 15 g agar, and 1 L distilled water, pH 7.0); D-1 medium, which is semi-selective for *Agrobacterium* spp. (30); Roy and Sasser’s medium, which is semi-selective for *R. vitis* (= *Agrobacterium* biovar 3) (35); 3DG medium, which is selective for *R. vitis* (= *Agrobacterium* biovar 3) (2); or grapevine-peptone (GP) medium (100 g macerated green shoot explants of grapevine ‘Pione’, 10 g peptone, 15 g agar, and 1 L distilled water). The plates were then incubated for 2 d at 27°C. ARK-1 was spotted onto five plates (one spot per plate) of each medium. To release antibiotics or plasmids from the bacterial cells (*i.e.*, to provide an insight into the antibiosis mechanism), the plates were then placed face down on a filter paper soaked with chloroform for 20 min. After chloroform had been evaporated, plates were inverted for 60 min to allow residual chloroform to escape. Five plates for each of the Ti strains, except VAT07-1n (Table 1), were misted with a cell suspension (about 10^7^ cells mL^−1^) of the strain as an indicator (sensitive) strain. The zone of inhibition was assessed 2 d after misting (Table 3). This assay was performed twice.

### *In planta* tumor inhibition assays

Tumor inhibition assays were carried out using methods we previously established (18, 19, 24). Grapevine seedlings (1-yr-old, *V. vinifera* L. cv. Neo Muscat) that were grown from seeds were prepared. The cell suspensions of tumorigenic strains VAT07-1 and AtC1 and nonpathogenic strain ARK-1 (Table 1) were prepared from 48-h-old cultures on PSA medium slants and adjusted to OD_600_ = 0.1 (corresponding to approximately 10^8^ cells mL^−1^), respectively. A cell suspension of tumorigenic strains VAT07-1 or AtC1 and strain ARK-1 was mixed in various combinations at cell ratios of 1:1. A 5-μL drop of a mixed cell suspension was dropped onto a needle-prick wound on the stem of a grapevine seedling. Five pots of grapevine seedlings (one plant per pot) each received 10 inoculations (*i.e.*, a total of 50 inoculations per treatment). The seedlings were grown in a greenhouse at 20 to 35°C with natural sunlight, and tumor formation was assessed two months later. This experiment was performed three times. The protective rate was defined as: Protection rate = 100% − ([% of tumor formation in mixed strain ARK-1 × 100]/[% of tumor formation by the pathogen only]).

### Data analysis

**Fig. 1:**
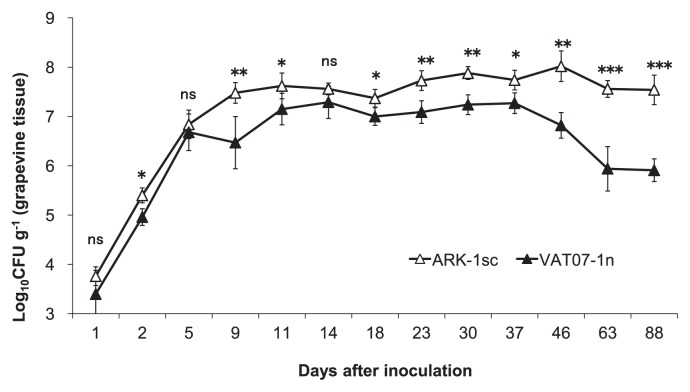
Populations of nonpathogenic strain ARK-1sc and tumorigenic strain VAT07-1n after inoculation on the shoots of grapevine seedlings at a 1:1 cell ratio. Data are means ± standard deviation for five seedlings. Significant differences at a given point in time were determined by the *t*-test (*P < 0.05; **P < 0.01; ***P < 0.001; ^ns^ *P* > 0.05).

**Fig. 2:**
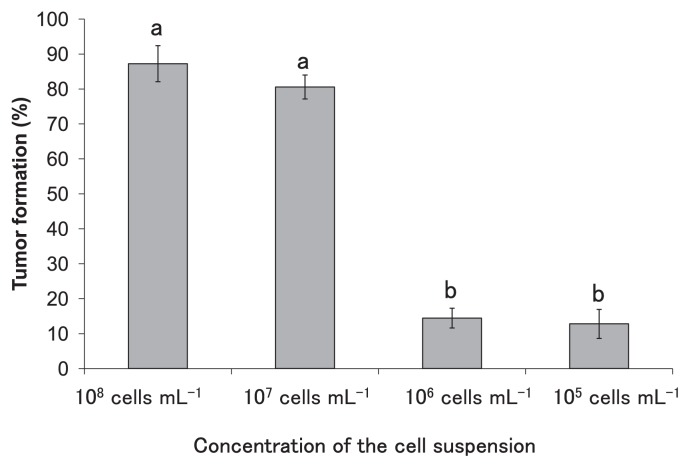
Dependence of tumor formation on each concentration of the cell suspension of tumorigenic strain VAT07-1n. Data are means ± standard deviation for three experiments. The different letters indicate a significant difference (*P* < 0.01) according to Tukey’s honestly significant difference (HSD) test of arcsine-transformed data.

Based on the description in Table 2 and Fig. 1, the *t*-test was used to compare the bacterial densities of ARK-1sc and VAT07-1n after the inoculation at a 1:1 cell ratio and also to compare population growth during the 88-d post-inoculation growth period. Based on the description in Fig. 2, Tukey’s honestly significant difference (HSD) test was used to compare each treatment. The *t*-test and Tukey’s HSD was performed in R software (The R Foundation for Statistical Computing, version 2.14.0). The Cochran-Mantel-Haenszel test was performed in XLSTAT software (Addinsoft Inc., Brooklyn, NY, USA) to compare the number of plants with tumors treated with ARK-1 with the number of the plants with tumors treated with the pathogen only (Table 4).

## Results

### Survival of ARK-1sc and VAT07-1n after the inoculation at a 1:1 cell ratio

Both mutants grew in St-CuSO_4_-PSA and Nal-PSA at wild-type rates in PSA medium (data not shown). The populations of ARK-1sc and VAT07-1n were similar, at approximately 10^7^ CFU g^−1^ (fresh weight) of the grapevine shoot, three months after the inoculation (Table 2). In the co-inoculation treatment, the population of ARK-1sc was approximately 5 × 10^6^ CFU g^−1^ (≈ 10^6.7^) of the grapevine shoot regardless of whether tumors formed. In contrast, the VAT07- 1n population was significantly higher in shoots with tumors (10^7^ CFU g^−1^) than in shoots without tumors (10^6^ CFU g^−1^).

### Population dynamics of ARK-1sc and VAT07-1n after the co-inoculation onto grapevine seedlings

The co-inoculation of ARK-1sc and VAT07-1n at a 1:1 cell ratio resulted in no crown gall formation over the 88-d study period (data not shown). The populations of ARK-1sc on the plants were significantly higher than those of VAT07-1n from 2 to 88 d after the inoculation (except at 5 and 14 d), and the magnitude of the difference increased after 37 d (Fig. 1). Colonization by ARK-1sc remained roughly constant at 4 × 10^7^ CFU g^−1^ (fresh weight) of the grapevine shoot for up to 88 d after the inoculation. In contrast, colonization by VAT07-1n never exceeded 1 × 10^7^ CFU g^−1^ of the grapevine shoot, and decreased to 1 × 10^6^ CFU g^−1^ of the grapevine shoot after 88 d.

### Dependence of tumor formation on the concentration of the pathogenic strain

Modeling experiments were performed to demonstrate the dependence of the incidence of tumors on variations in the titers of the pathogenic strain between 10^7^ and 10^6^ cells mL^−1^ to confirm that the decrease provided by ARK-1 was the main reason for the observed effect. The proportions of tumors that formed on the grapevine stems, which were inoculated with cell suspensions of VAT07-1n at 10^6^ and 10^5^ cells mL^−1^, were significantly lower than those inoculated with 10^8^ and 10^7^ cells mL^−1^ (Fig. 2). The mean proportion of tumors that formed on the grapevine stems inoculated with 10^6^ cells/mL was under 15%, while that of the grapevine stems inoculated with 10^7^ cells mL^−1^ was over 80% (Fig. 2).

### *In vitro* antibiosis assay

Although all combinations of ARK-1 and indicator strains, except for *R. radiobacter* (Ti) strain AtC1, resulted in zones of inhibition on PSA and King’s B medium, ARK-1 did not inhibit any of the indicator strains on YMA, NA, D-1, Roy and Sasser, 3DG, or GP media (Table 3). These results were identical in both repetitions of this experiment.

### *In planta* tumor inhibition assays

Tumors were observed on the shoots of the grapevine seedlings in all three independent experiments. On grapevine seedlings, a 1:1 cell ratio of strain ARK-1 to Ti strain VAT07-1, which was sensitive to the antibiotic activity of ARK-1, significantly suppressed the incidence of tumors (*P* < 0.001) in stems relative to the only VAT07-1 control, and the protection rate was 83.9% (Table 4). A 1:1 cell ratio of strain ARK-1 to Ti strain AtC1, which was not sensitive to the antibiotic activity of ARK-1, significantly suppressed the incidence of tumors (*P* < 0.001) in stems relative to the only AtC1 control, and the protection rate was 93.7% (Table 4).

## Discussion

In the co-inoculation assay, the populations of VAT07-1n were approximately 10^7^ cells g^−1^ (fresh weight) in shoots with tumors and 10^6^ cells g^−1^ in shoots without. These results demonstrated that the tumorigenic bacterial population co-inoculated with ARK-1sc was ten-fold less than the population without this ARK-1sc, and this difference was significant; thus, ARK-1sc significantly inhibited the growth of the Ti strain.

These results confirmed the expected inhibition of tumor formation by ARK-1. However, ARK-1 did not completely eliminate the formation of tumors on grapevines, as shown in previous greenhouse and field trials in which the incidence of crown gall disease during treatments with ARK-1 decreased by 15% (20, 27). When ARK-1 does not inhibit the growth of tumorigenic bacteria in plants, Ti strains may reach population densities of up to 10^7^ cells g^−1^ of the grapevine shoot, leading to the development of the symptoms of crown gall. In the present study, the population of VAT07-1n after the co-inoculation with ARK-1sc reached 10^7^ cells g^−1^ in shoots that formed tumors. At grapevine wound sites co-inoculated with ARK-1sc and VAT07-1n, the population of ARK-1sc remained roughly constant at approximately 10^7^ CFU g^−1^ of the grapevine shoot up to 88 d after the inoculation, whereas the population of VAT07-1n never exceeded the population of ARK-1sc, and decreased to <10^6^ CFU g^−1^ (4 × 10^5^ CFU g^−1^). Moreover, the inoculation with 10^6^ cells mL^−1^ of VAT07-1n induced significantly fewer tumors on the grapevine stems than that with 10^7^ cells mL^−1^. These results suggest that the mechanism underlying inhibition by ARK-1 may be bacteriostatic rather than bactericidal. ARK-1 began to suppress the growth of the Ti strain significantly by 2 d after the inoculation. This result indicated that ARK-1 controlled the pathogen population at the grapevine wound site. The degree of inhibition of the growth of the Ti strain by ARK-1 changed over time. In contrast, F2/5 did not inhibit the survival or growth of *R. vitis* (Ti) strains at grapevine wound sites (22).

Wang *et al.* (39) reported that an antibacterial compound named Ar26 produced by nonpathogenic *R. vitis* strain E26 inhibited the growth of some Ti strains of *Rhizobium* on culture plates. A previous study (20) showed that the antibiotic activity of nonpathogenic *R. vitis* VAR03-1 may be one of the factors responsible for the control of apple crown gall. On the other hand, Burr and Reid (6) reported that the biological control of grapevine crown gall by nonpathogenic *R. vitis* F2/5 was not associated with the production of agrocin or competition for attachment sites on grapevine cells. The antibiosis assay in the present study showed that the antibiotic activity of ARK-1 inhibited three strains of *R. radiobacter* (Ti), three strains of *R. rhizogenes* (Ti), and four strains of *R. vitis* (Ti) on PSA and King’s B media, but did not inhibit these strains on the other media (Table 2), suggesting that antibiotic activity depended on the medium. The composition of the GP medium, which contained macerated grapevine shoot explants and peptone, was the closest to that of natural grapevines. ARK-1 did not inhibit the growth of *R. vitis* (Ti) strains on GP medium (Table 3), which suggests that ARK-1 may not have antibiotic effects on grapevines. Moreover, if antibiotic activity blocks the bacterial synthesis of some nutrients, which can be present in the culture medium, bacteria may use that nutrient to continue to grow.

*R. radiobacter* (Ti) strain AtC1 was insensitive to the antibiotic activity of ARK-1, and ARK-1 did not inhibit its growth on PSA or King’s B media. Nevertheless, ARK-1 suppressed crown gall formation on grapevines caused by AtC1, indicating that the control mechanism of ARK-1 may depend on factors other than antibiotic activity. In addition, ARK-1 did not reduce the incidence of tumors on the grape-vine shoots when ARK-1 cells were dead or when only the filtrate of the PS broth culture was used (24), which supports this result and suggests that ARK-1 antibiotic activity may not be the main control factor *in planta*.

Creasap *et al.* (12) showed that at least one of the *luxR* homologs, *aviR*, as well as the *clpA* homolog, was involved in biological control by strain F2/5 and that one or more necrosis mechanisms (*e.g.*, the hypersensitivity reaction) may be related to biological control. Kaewnum *et al.* (17) demonstrated that two regulatory systems, the quorum-sensing and caseinolytic protease (clp) systems, were associated with the biological control mechanism of strain F2/5. Although F2/5 induces necrosis and controls crown gall on grapevines, but not on other plant species, ARK-1 did not induce necrosis on grapevines (27). ARK-1 is a different type of antagonistic strain from F2/5. Thus, the expression of the *luxR*, *aviR*, and *clpA* genes needs to be investigated in ARK- 1.

## Conclusion

ARK-1 reduced the pathogen population at grapevine wound sites, but antibiotic activity was not the main mechanism. These results provide an insight into the mechanism responsible for biological control, and suggest that ARK-1 may have a unique, previously unreported mechanism responsible for its control of crown gall. Further studies are warranted to elucidate this mechanism in more detail.

## Supplementary Information



## Figures and Tables

**Table 1:** Bacterial strains used in this study

Bacterial strain	Pathogenicity^a^	Opine type	Description (supplier)
*Rhizobium radiobacter* (Ti) (= *Agrobacterium tumefaciens* (Ti), *A. tumefaciens* biovar 1)			
ARAT001	Ti	Nopaline	Isolated from galled apple trees in Japan (K. Suzaki) (25)
CH3	Ti	Unknown	Isolated from galled chrysanthemums in Japan (T. Takikawa) (20)
CH5	Ti	Unknown	Isolated from galled chrysanthemums in Japan (T. Takikawa) (20)
AtC1	Ti	Agrosinopine, Mannopine, Octopine	Isolated by M. Ohta from galled chrysanthemums in Japan (T. Takikawa) (18)
*R. rhizogenes* (Ti) (= *A. rhizogenes* (Ti), *A. tumefaciens* biovar 2)			
ARAT002	Ti	Nopaline	Isolated from galled apple trees in Japan (K. Suzaki) (25)
NEAR8	Ti	Unknown	Isolated from galled apple trees in Japan (K. Kondo) (25)
NEAR11	Ti	Unknown	Isolated from galled apple trees in Japan (K. Kondo) (25)
*R. vitis* (Ti) (= *A. vitis* (Ti), *A. tumefaciens* biovar 3)			
G-Ag-27	Ti	Vitopine	Isolated from galled grapevine trees in Japan (H. Sawada) (24)
MAFF211674	Ti	Unknown	Isolated from galled grapevine trees in Japan (MAFF^b^) (24)
VAT07-1	Ti	Nopaline	Isolated from galled grapevine trees in Japan (24)
UK-2	Ti	Octopine	Isolated from galled apple trees in Japan (T. Misawa) (24)
9-3-4	Ti	Unknown	Isolated from galled grapevine trees in Japan (This study)
VAT07-1n	Ti	Nopaline	Nalidixic acid-resistant mutant of strain VAT07-1 (This study)
Nonpathogenic *R. vitis* (=Nonpathogenic *A. vitis*, *A. radiobacter* biovar 3)			
ARK-1	N	…	Isolated from a nursery stock of grapevines in Japan; biological control agent for crown gall (24, 27)
ARK-1sc	N	…	Streptomycin- and copper sulfate-resistant mutant of strain ARK-1 (24, 27)

a Ti: Tumorigenic. N: Nonpathogenic.

b MAFF: Ministry of Agriculture, Forestry and Fisheries, Tsukuba Ibaraki, Japan.

**Table 2:** Populations of nonpathogenic strain ARK-1sc and tumorigenic strain VAT07-1n after inoculation at a 1:1 cell ratio on grapevine seedlings^a^

Strain	Tumor formation	Log_10_ CFU g^−1^ (fresh weight) grapevine shoot^b^	

		ARK-1sc	VAT07-1n
ARK-1sc	−	7.0 ± 0.1	nd
VAT07-1n	+	nd	7.1 ± 0.1
ARK-1sc + VAT07-1n	−	6.7 ± 0.1***	6.0 ± 0.5
ARK-1sc + VAT07-1n	+	6.7 ± 0.1*	7.0 ± 0.1

a Data are means of 10 samples of grape shoots. Values are means (after log_10_ transformation) ± standard error.

b Significantly different from the VAT07-1n population (*t*-test; ***P < 0.001, *P < 0.05).

c nd; no detection.

**Table 3:** *In vitro* antibiosis assay of nonpathogenic *Rhizobium vitis* strain ARK-1 against *Rhizobium* Ti strains

Indicator strain	Formation of an inhibition zone^a^ on the medium plate							

	PSA	King’s B	YMA	NA	D-1	Roy and Sasser	3DG	GP
*R. radiobacter* (Ti)								
ARAT001	++	++	−	−	−	ng	ng	ng
CH3	+	+	−	−	−	ng	ng	ng
CH5	+	+	−	−	−	ng	ng	ng
AtC1	−	−	−	−	−	−	−	−
*R. rhizogenes* (Ti)								
ARAT002	+	+	−	−	−	ng	ng	ng
NEAR8	+	+	−	−	−	ng	ng	ng
NEAR11	+	+	−	−	−	ng	ng	ng
*R. vitis* (Ti)								
G-Ag-27	++	++	−	−	−	−	−	−
MAFF211674	++	++	−	−	−	−	−	−
VAT07-1	++	++	−	−	−	−	−	−
UK-2	++	++	−	−	−	−	−	−

a Relative size of the inhibition zone (mm): − = no inhibition, 10 < + ≤ 15, 15 < ++ ≤ 20, ng = indicator strains did not grow.

**Table 4:** Effects of the co-inoculation with nonpathogenic *Rhizobium vitis* and tumorigenic strains at a 1:1 cell ratio on grapevine seedlings. Strain VAT07-1 was sensitive to antibiosis by ARK-1; strain AtC1 was insensitive

		Against Ti strain sensitive to antibiosis activity				Against Ti strain insensitive to antibiosis activity			
			
Strain	Experiment	No. of plants	No. of inoculations^a^	Proportion of tumor formation (%)^b^	Protection rate (%)	No. of plants	No. of inoculations^a^	Proportion of tumor formation (%)^b^	Protection rate (%)
ARK-1	1	5	50	8.0		5	50	2.0	
	2	5	50	18.0		5	50	0.0	
	3	5	50	16.0		5	50	12.0	
	Mean			14.0***	83.9			4.7***	93.4
Only pathogen	1	5	50	92.0		5	50	66.0	
	2	5	50	82.0		5	50	68.0	
	3	5	50	86.0		5	50	80.0	
	Mean			86.7				71.3	

a Ten inoculations per plant.

b Means followed by asterisks differ significantly between the ARK-1 treatment and sterile water treatment (Cochran-Mantel-Haenszel test, ***P < 0.001).
