# Supplementary figures and images for: Reduction in Pathogen Populations at Grapevine Wound Sites is Associated with the Mechanism Underlying the Biological Control of Crown Gall by Rhizobium vitis Strain ARK-1
Source: Microbes Environ. 2014 Jul 31;29(3):296–302. doi: 10.1264/jsme2.ME14059 (PMC4159041; doi:10.1264/jsme2.ME14059)

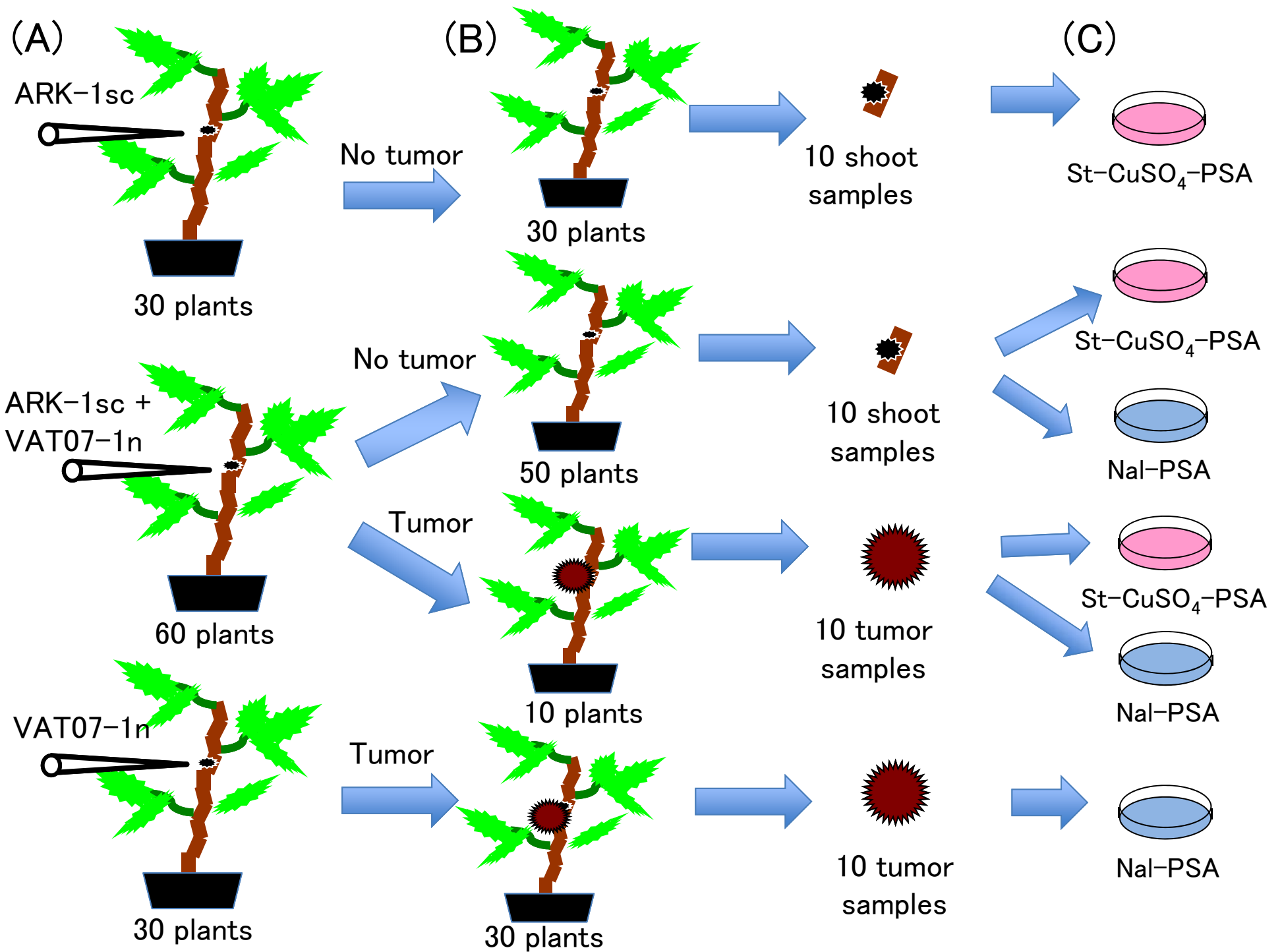

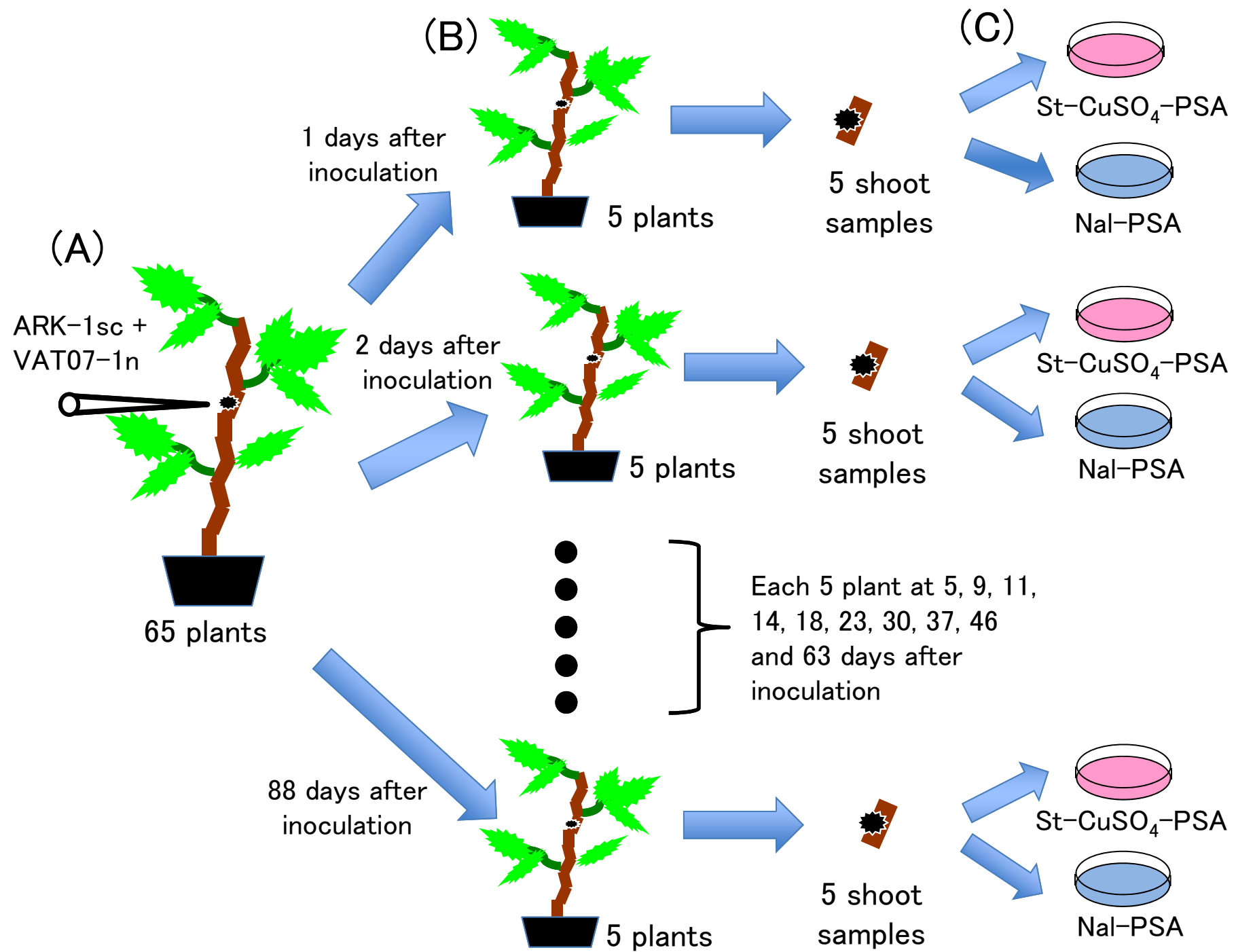

Supplement: Supplementary file 1 [file 29_296_s1.pdf]
